# Commentaries on Some Doctrines of a Dangerous Tendency in Medicine, and on the General Principles of Safe Practice

**Published:** 1843-04

**Authors:** 


### Art. XIV.

*Commentaries on some Doctrines of a dangerous tendency in Medicine, and on the general principles of safe Practice.* By Sir Alexander Crichton, m.d. f.r.s. l.s. & g.s., &c. &c.—*London*, 1842. 8vo, pp. 283.

These Commentaries constitute a review of medical opinions and practices prevailing during a long period of time; and considering that the author states in his preface that he has attained the age of seventy-nine, and when it appears that his period of professional observation exceeds considerably half a century, it must be admitted that there are strong arguments for his competence to the office which he has assumed. But when we discover, moreover, which the perusal of even a few pages enables us to do, that age, whilst it has abated nothing of the vigour of his intellect, has added prudence and sobriety to its exercise, and when it is likewise manifest that he has been a reader, observer, and thinker, in various climates, and under circumstances the most favorable to such pursuits, our expectations of pleasure and profit from the office of reviewing his work become very much exalted. The age which Sir Alexander Crichton has attained, and the eminence on which he stands, place him beyond the hopes and fears of professional life, and are a security for his addressing the public solely on the legitimate ground of his having something to say to it which he considers deserving of its attention. We cannot contemplate without respect a man, arrived at an age when repose is to most so seductive—and all would be excused for yielding to the seduction—who musters his strength to impart to others those truths which he has employed so many years in collecting. He is not satisfied, like the contemplative philosopher in Lucretius, to exult in his own exemption from error, whilst he sees others darkly straying from the truth; but he extends to them the torch which he thinks likely to guide them in its paths.

These prepossessions, however, ought not to influence our critical judgment, nor shall they do so. Sir Alexander's work must be appreciated at its intrinsic value, and not for the venerable age, or high respectability of the party whence it has emanated; and we shall now proceed to review its contents, and to comment upon them, bearing ever in mind the objects of the author, who does not profess to deal in novel views, but, from contemporary opinions and practices, to select and recommend the most worthy.

*The functions of the heart and arteries.* The first commentary is on a physiological question,—the functions of the heart and arteries. The doctrines compared are, that originally promulgated by Haller, and subsequently adopted by various eminent physiologists, according to which the blood, after its expulsion from the heart, is propelled in the arteries, and especially in the capillaries, by a regular succession of muscular contractions and relaxations of these vessels, similar to the systole and diastole of the heart, and that published by Dr. Thomas Young, in the Philosophical Transactions for 1809, denying such action to arteries, though admitting the existence of a muscular coat in their structure, the effect of which he considers to be merely an increase of the elasticity of the artery. The former part of Sir Alexander's commentary is to confirm the view taken by Dr. Young, though with some modifications, the nature of which is not always very clearly explained. He is of opinion that every phenomenon of the circulation of the blood in its usual or normal course, which depends on arterial pressure, may, indeed, be explained by the elasticity of the arteries. But, although for the wisest ends, these vessels are very insensible to nervous excitement and impressions, and consequently are not exposed, like some other organs, to be agitated and disturbed by the passions, yet, like most of the living structures of our complicated frame, they may, under certain circumstances, be influenced by affections of the brain. In no other way, he remarks, can we account for either the turgescence or collapse of portions of the blood-vessels from mental causes, or from certain stimulants and sedatives, the first action of which is on the stomach.

Those well-known partial deviations from the ordinary tenor of the circulation, from mental or other causes acting on the nervous system, certainly appear utterly irreconcileable with the assumption that the sole moving powers of the blood are the central organ, and mere elasticity of the arteries. This elasticity, however, Sir Alexander tells us (p. 31) is of a vital nature, and may be increased or diminished by the exaltation or diminution of cerebral influence. We have certainly always regarded elasticity as a quality depending for its existence and degree on the nature or texture of the substance endowed with it; and we cannot help regarding the application of the term to a quality admitting of sudden variations from mere emotion, or causes acting sympathetically, which cannot have any influence on the texture of arteries, as calculated to introduce confusion into our language and reasonings on the subject of the circulation.

Not doubting that the weaker side of the controversy is that embraced by our author, and believing that there is no little confusion, at least, in his expression of the modifications he would introduce into the doctrine which he professes to support, we question whether his error does not consist rather in phraseology than in fact. His own modifications of the doctrine, as we understand them, would convert the property which he terms elasticity into a capacity of action having a much closer relation to vital dilatation and contraction than to the merely physical quality ordinarily designated by this term. Our limits will not allow us to enter upon the consideration of this subject at present; but we would recommend to the notice of Sir Alexander Crichton, and our readers, the experiments and observations in reference to this point by Sir Charles Bell, in the volume of Essays published just before the author's lamented death. Although containing less novelty, perhaps, than their author imagined, they afford a completer and more intelligible exposition of the questions at issue than is to be found in any recent work.

Our author has two sections on *Exercise* and *Inaction*, to his instructions on which subjects his views on the circulation are preliminary. We think his practical precepts valuable, but their truth so entirely independent of all theories of the circulation, that we cannot see the necessity for the hypothetical disquisition which serves as their preface. He is friendly to regular and opposed to violent exercise, especially after a full meal,—as who is not? Observation, he tells us, among agricultural labourers and the poorer classes generally, have shown him that aneurism of the aorta, and dilatation of the left ventricle of the heart, are frequent diseases of this class ; and he thinks great muscular efforts, especially after taking food, a very frequent cause of them, the muscular exertions throwing more blood on the central organ of circulation than it can dispose of. Of the frequency of the diseases in question, especially of the heart-affection, no one can feel a doubt whose opportunities of observation have been considerable, or who is acquainted with Dr. Clendinning's statistical details on the subject; and we think there is much reason for ascribing their origin to violent exertion. We are the more inclined to the latter opinion from having observed the great frequency of diseases of the heart among sailors, a class in which violent exertion is of constant occurrence. In the following passage, Sir Alexander indicates, and we think with truth, an error in the management of exercise by a different class :

"A common error is frequently committed, from ignorance or from false notions, by those who have a full command of their time and pursuits. Many unemployed men of wealth imagine that if they dedicate several hours, consecutively, in the early part of the day, to a fox-chase, to a stag-hunt, to a game of cricket of many hours' duration, or a tennis, or in fencing or any other gymnastic exercise, they have done that which contributes much to health, at least for one day; judging perhaps by the keen appetite which so great a waste of solids and fluids occasions. The consequence is, that the remainder of the twenty-four hours is apt to be spent in nearly complete inaction, and hurtful indulgence. Many strong and healthy young people may unquestionably derive benefit from such exercises in the open air, but that many others are injured by it is certain, for the reasons assigned, as well as for others I am about to mention." (Crichton, p. 35.)

Our author has a passage on the effects of severe muscular exertion in young and delicate persons whose thoracic organs are imperfectly developed in proportion to the aorta. To the affection produced by this cause in persons so constructed, he gives the name of asthenia of the heart, and he thus describes it:

"Its most characteristic symptoms are pains in some parts of the spinal column, a sense of great languor and fatigue, and more or less muscular weakness in the lower extremities, the pulse being generally quicker and smaller than usual, together with some disturbance of the digestive organs." (p. 38.)

This is a condition abundantly familiar to us and most medical men; but we never thought of ascribing it to asthenia of the heart exclusively. The whole system is in a state of asthenia, and we would say, that if the defect of energy were more manifest in one part of the system than another, it was in the nervous centres. This preponderance is indicated by incapacity of sustained attention and any considerable mental exertion, by the feebleness of the lower extremities to an extent to cause the patient to trip in walking, and we would add, too, by the pain in the course of the spine, so properly enrolled among the characteristic symptoms by Sir Alexander Crichton. He questions, too, its origin from excessive exertion, having generally found it to occur in young persons leading sedentery lives, in students for instance, and young persons engaged in counting-houses, and lawyers' offices. Though differing from our author, in some degree as to the nature, and certainly as to the cause of the disease, we concur in his method of treatment, which he tells us should consist in corporeal and mental rest, a nutritious diet of easy digestion, given to the extent and as often as the patient can bear it, small doses of quinine and sulphuric acid or vinum ferri, and an opiate at bed-time, should sleep be wanting. We concur, as we have said in all this with the exception of the corporeal rest, having found a gradual progression of exercise, according to the following scale, generally to promote recovery,—gestation in an open carriage, gentle rides on horseback, walking.

*Typhus fever.* We now reach the second and most important commentary, that on the doctrines and treatment of continued fevers of a typhoid character. Sir Alexander's extensive opportunities of observing disease, and acquaintance with physicians and their mode of thinking and acting in various countries, render this section of his work at once instructive and amusing. It appears that he proceeded to Russia in 1804, when Brunonianism, under the name of the "*Erregungstheorie*" (excitement-theory) and in its worst form had taken possession of the minds of Germany. To these dreamers succeeded in 1810, a race of sophists, of a still more intractable character, *the philosophising physicians of nature* (die natur-philosophischen Aerzte) issued from the schools of Schelling, Kilian, and Oken. It was the duty of our author to superintend and direct the practice in the various medical establishments of the Russian empire, and the majority of his subordinates were Germans deeply imbued with one or other of these pestilent fancies. With the disciples of Brown he could reason; but not so with his philosophising coadjutors, for he could understand neither their language nor their definitions. This will be deemed no serious imputation on the perspicacity of the worthy knight, when it is found that he was treated to such *lucid* matters as the following: "Light is the separation of that which is absolute; it is the primitive action of pure activity; it is the animating soul of ether by which it is placed in polar tension." This is the definition of light by Schelling. The following definitions of disease are from the same high authority and his disciples: "Disease is a change of the dimensions of human organism;" or, "it is a disproportion between organic activity and organic structure;" or again, "it is the opposition of the special or specific quality of *organismus* united to the *idea* of life;" or finally, "it is the reigning difference of individuality and universality." One of these worthies seems to have understood his real vocation when he replied to Sir Alexander, who, it would seem, was labouring (of course in vain) to inspire him with a little common sense: "Sir, we are taught to think deeply, and to write on the philosophy of nature and of medicine, but not to practice it. Procure a professor's chair for me; that is my vocation." (p. 63.)

We think it a matter of regret that the successors of these *philosophisers* have not been equally cognizant with the would-be professor, of their real calling. To dream, and publish dreams, are occupations often amusing and generally innocuous; but dreams reduced to action in the form of homœopathy and hydropathy, involve the health and lives of thousands.

We do not deem it necessary to follow our author through his criticism of Rasori's *diathesis di stimulo* and *di contra-stimulo*, and the extravagant practice which resulted from them. We should quote largely from this section of the work which treats of these matters, did we think there was a possibility in the present era of any patient in any country losing in nine days, fifteen pounds of blood, and swallowing 220 grains of digitilis, as did one unhappy victim of Rasori's doctrine. But as we have evidence in the recent history of hydropathy* that the reign of error and dangerous error is not yet at an end, we would recommend, if in any corner of the Old or New World, doctrines so absurd, leading to practices so murderous, as those of Rasori still linger, that *there* the commentary included between the 63d and 77th page of Sir Alexander's work should have careful perusal and consideration.

A very important portion of this commentary is that in which what may be called the French doctrine of fever, and the practices founded by some upon it, is examined. The opinion that what were by Pinel and physicians of this country considered as essential fevers, are dependent for their existence on a lesion, generally of an inflammatory nature, in the alimentary canal, is still so prevalent in a modified form, at least in France, that we cannot be in error in considering it the reigning doctrine there, especially when we find the same doctrine entertained, with some slight modifications, by Louis and Cruveilhier. Sir Alexander bestows on these distinguished parties, and especially on Andral, much praise for their pathological discoveries, but expresses his regret that such discoveries should have tended to injure rather than improve practical medicine; but this must always occur, he remarks, when morbid appearances are incorrectly interpreted.

Not only, however, does he differ from these French authorities as to their practical inferences, but questions the universality at least of the facts whence they are deduced, quoting Dr. Little of Belfast, to show that of sixty fatal cases of fever he did not find one with signs of *acute* inflammation of the mucous membrane of the stomach and intestines. The question, however, does not regard the existence of acute inflammation of the stomach and intestines excepting perhaps with Broussais; with the other writers it relates to the well-known affections of the plates of Peyer, and the glands of Brunner, constituting conjointly what Bretonneau has called *dothinenteritis*, and there can be now no doubt that this, though less frequently, we believe, in this country than some others, is a very general accompaniment indeed of typhus.

But however general it may be, even allowing that it should be found, as estimated by Andral, in ninety-eight of 100 cases of typhus, and we are convinced that in the typhus of Britain it would not be found in so high a ratio, though we should deem it an important element, we yet could not consider it the essential constituent, or (to use a phrase now nearly and deservedly obsolete,) the proximate cause of the disease,

* In our remarks on hydropathy we would wish it to be distinctly understood that we have no intention of questioning the benefit conferred in certain chronic disorders, the fruit of sloth and indulgence, by the cold water process of Vincent Priessnitz and the hygiènic discipline with which it is associated. But with whatever discretion the strong sense of the author of this system may lead him to restrict it to certain chronic derangements of function to which alone it can be applicable, we have abundant evidence that his followers in this country, not much more erudite and certainly less wise than their master, lavish it indiscriminately on acute and chronic—sthenic and asthenic disease, with what result coroners' inquests will ere long decide. Would any medical man choose to have inflammation of the lungs or peritoneum in his own person treated exclusively by wet cloths ove the chest or abdomen?

since it is not, even on the showing of Andral and Bretonneau, its universal accompaniment. That it is an important element is manifest from one of Andral's cases, (and many similar examples might be adduced,) in which *dothinenteritis* proceeded so far that the intestine was perforated and death ensued; that it is one of frequent occurrence is admitted on all hands; but how it should influence our treatment of the disease has not yet been shown. M. Andral, it would seem, was led by his knowledge of the pathological condition to bleed rather largely in some cases of fever. In one of these cases three bleedings of twelve ounces each were performed. The case was fatal on the ninth day, and on examination the small intestines to the extent of about two feet immediately above the ileo-cæcal valve, were found studded with five large oval patches of a grayish-red colour. Between the patches small pustules, some of which were red, others of a grayish-white colour, were detected; but the rest of the mucous membrane was pale. M. Andral is too judicious a man to continue in a wrong course; and he has stated that in some cases, in which an aggravation of symptoms occurred after blood-letting, a sudden amelioration took place when cinchona and other tonics were administered. The cases, however, treated by the former means furnish ground to Sir Alexander Crichton for considerable comment.

He illustrates his opinion of the inexpediency of the depletory treatment of typhus by two other disorders bearing more or less analogy to this affection, the malignant thrush fever, occasionally seen in this country, and common in Holland and Brabant; and scarlatina. In the former of these complaints, there are inflammation and thrush in the mouth and fauces, pains in the abdomen, vomiting, frequent stools in which aphthous crusts are visible, and an appearance of aphthæ about the anus. There is inflammation apparently through the whole course of the digestive mucous lining; yet bleeding is never employed for it, but success follows a different line of treatment, namely, deterging the intestinal tract by a combination of antimonials and mild purgatives, and by supporting the strength of the patient, as much as the heat, headach, and general febrile symptoms permit, by mild nutritious drinks, such as barley-water, weak chicken or beef tea, then by wine, and afterwards by a decoction of cinchona combined with small quantities of muriatic acid. On descanting on the other disease selected for comparison with typhus, scarlatina anginosa gravior, Sir Alexander remarks that the membranes of the mouth, fauces, nose, œsophagus, and meatus auditorius, are in a state of high inflammation, and then asks, whether it enters into the head of any good practitioner to attempt to cure this inflammatory action by taking away forty ounces or even five ounces of blood from the arm! or, to apply leeches to the neck and throat, to relieve the swelling? The former of these queries we answer at once in the negative; but to the latter we reply, that we are constantly in the habit of applying leeches to the neck and throat to relieve the swelling, and this too, with benefit to the patient, which, we trust, will relieve us from the imputation of being bad practitioners for so doing.

We think that arguments against bleeding in typhus, provided that such bleeding is performed for the purpose of subduing inflammation of the muciparous glands and mucous membrane, of a more cogent nature than those deduced from the analogy of thrush and scarlet fever might easily have been found. All experienced physicians must have observed in cases of bronchitis, of muco-gastritis and muco-enteritis existing as independent diseases, how little beneficial influence is exerted by general bloodletting on inflammation of mucous membrane and its associated glandular apparatus. What a contrast does the paucity of effect produced present to the result of the same measure employed for inflammation of a serous membrane, in pleurisy for instance?

Nature's principle of curing typhus, says our author, is the same as that by which the paroxysm of an intermittent is terminated; she reduces the quantity of circulating fluids until she brings about an equilibrium between them and the enfeebled moving powers.

This certainly sounds like an argument in favour of bloodletting; but Sir Alexander points out, and with truth, the distinction between the excrementitious evacuation by sweat in intermittent, and the withdrawing of a portion of the entire and vital blood. He admits, however, of bleeding, not to a greater amount than two ounces, in cases of suspected inflammation, or threatened congestion. We certainly think the bleeding here mentioned not likely either to subdue the inflammation, or prevent the congestion. The question of bleeding in typhus seems to us to be rather dogmatized upon by Sir Alexander, than examined on sound therapeutic principles. His arguments against it appear to us too universal, and on the whole overcharged. Whether this has arisen from his living on the continent, having witnessed exclusively a low sunk form of typhus in which evacuants would have proved especially pernicious, or, from his having been appalled by the sanguinary exploits of some rabid disciple of Broussais, we know not; but it has certainly arisen from too limited a view of the disease and its treatment. Rules of treatment cannot be laid down thus universally. They should be framed conditionally, and applied according to the precept of the most sagacious of physicians, Sydenham, "*perspecto genio morbi.*" Now this "*genius morbi*" differs very materially indeed in different epidemics of typhus; of which we will give an example, on indubitable authority, from the practice of a physician whom our author frequently refers to and quotes, the late Dr. J. Clark of Newcastle. This eminent man, in an epidemic of typhus which prevailed in Newcastle and its neighbourhood, in the latter end of the last century, employed his well-known practice of giving bark with cinnamon water and brandy, with such success that it procured for him a reputation perhaps unparalleled among provincial physicians. But in a subsequent epidemic in the same district, the same means, applied by the same hands, were as signally unsuccessful as in the former case they had been prosperous. The worldly circumstances of a patient, and the locality in which he resides, influence very much the character of the disease and the mode of its correct treatment. Sir Alexander Crichton quotes largely from the writings on fever of the Dublin physicians, and in a spirit of well-merited eulogy. But he would acknowledge that many practices must be resorted to in the case of the squalid denizen of the liberties of Dublin, which would be inapplicable to that of the well-fed inhabitant in the middle or upper ranks of life of a province in England.

Though holding the opinion that a more extensive view of typhus in England might have mitigated a certain tone of dogmatism displayed when he treats of bloodletting, we bear willing testimony to the excellent judgment manifested in our author's commentary on the other remedies of the disease. He speaks with well-deserved praise of the effect of belladonna, first employed, we believe, by Dr. Graves, though the original suggestion of its employment seems to have proceeded from Dr. Corringan, in that dangerous form of brain-affection in typhus, in which the pupil is so contracted as to form what Dr. Graves in his emphatic language calls the *pin-hole* pupil. His remarks on the employment of antimony, of the warm-bath, and of the cold aspersion and affusion of Dr. Currie, and the circumstances limiting their applicability, all denote the experienced and prudent physician.

When meteorismus is accompanied by pain on pressure rendering it probable that inflammation of the intestines exists, he recommends the application of blisters, combined with the judicious administration of calomel in small doses united to antimonial powder, or ipecacuanha. These means we consider to be very judicious, and we would add that we have found the symptoms mentioned to be very general accompaniments of *dothinenteritis*, and very frequent precursors of intestinal hemorrhage. When this has occurred we have observed that the internal employment of acetate of lead has not only allayed the hemorrhage, but has appeared to be beneficial to the inflammation and ulceration on which it was dependent. We do not quite concur in our author's fondness for emetics in fever, especially in the bilious synochus of this country, which he very aptly compares to the bilious remittent which prevails along the shores of the Mediterranean. Now in this latter disease we have ever observed a degree of gastric irritation counter-indicating the employment of emetics, and pernicious effects have even to us appeared to result from them.

Sir Alexander's disquisitions on the doctrine of fever are learned and interesting; and if we do not give them in detail it will be understood that it does not arise from any disparaging appreciation of their merit, but for want of space. He adopts Liebig's theory of the action of contagion and other animal poisons, the poison of bad sausages for instance, and expresses it in the following terms: "If the exciting agents of chemical metamorphosis be a compound body, which is in the act of decomposition, it will reproduce itself *ad infinitum*, provided the compound body on which it acts contains elements fitted for such an end." Sir Alexander thinks that it is the blood and especially its serum, in which the metamorphosis chiefly takes place in the case of typhus. To this conclusion regarding the serum he is led by considering that the low state of vitality of this fluid presents little resistance to the action of the poison, contagion. The influence of this poison is next manifested in the diminution of vital force in the brain, and consequently in the organs of circulation, those of digestion and of secretion, and so long as any specific virus or contagion finds materials in the blood, which, by the law of chemical transformation, it can set in motion and gradually convert into a compound *ejusdam generis*, so long does the disease continue. The disorder ceases where such elements have all undergone the transformation or are exhausted.

It must be obvious, since we have no means of destroying this assimilative power of the poisonous agent, that our practice in fever can amount at best only to a rational empiricism, and that we must learn from experience how best to combat its effects. It is gratifying to be consoled in this state of apparent impotency by the high authority of Liebig who informs us that methods, the results of ages of experience, are such, that the most perfect theory could hardly have pointed them out more acutely or justly than has been done by the observation of sagacious practitioners.

*Insanity.* The third commentary is on insanity. It contains not much that is new, but much that is judicious. The author's definition of the disease stands thus :

"Insanity is a disease of the brain which causes the patient, while awake, to mistake the phantoms and operations of imagination for realities, which, consequently, become the motives of his discourse and actions, while at the time there is an absence of any bodily disorder that can account for the phenomena." (p. 165.)

We think that in this definition more things are comprehended than insanity. But for its first proposition ("insanity is a disease of the brain"), which is manifestly hypothetical, it would comprise the error of the touchy man, who feels and resents as an affront what was not intended, nor were there *reasonable* grounds for supposing it to be intended, as such; but all who have tried having felt the extreme, perhaps the insurmountable, difficulty of framing a definition of the disease at once comprehensive and exclusive, we are not disposed to be critical on Sir Alexander's efforts.

He bestows much commendation on MM. Foville and Delaye for their investigation of the condition of the brain in this disease, expressing the opinion that this organ has never been satisfactorily examined but by them. M. Foville is especially praised for his discovery of the red colour and manifestly augmented vascularity of the gray or cineritious matter in fatal cases of acute mania. In chronic cases only has this writer found cohesion of the membranes of the brain to its cortical substance.

The plan of treatment, however, which the condition of the organ in acute mania would seem to indicate, that of bloodletting, is one of which he does not approve. He demurs to the conclusion drawn by Foville from his anatomical observations, that we are justified in employing the most active antiphlogistic means at the commencement of the disease, to subdue the inflammation. On this Sir Alexander remarks, that there are a variety of congestive states of blood-vessels designated inflammation, though all of them are perfectly distinct from this state. We are of opinion that congestion, or hyperæmia, frequently exists without that effusion, or those new formations which are of the very essence of inflammation,—that, though congestion is a constituent of inflammation, it is often found in the absence of this condition. In stating, however, this familiar truth, we must guard ourselves from accordance with the classification of our author, who ranks syphilis, variola, and vaccine, and some other diseases, with congestive states of blood-vessels. The diseases we have named certainly display, in new formations, the characteristics of inflammation; but the causes whence these specific inflammations proceed invest their whole course with a peculiarity, distinct in each disease, and discriminating them not only from each other, but collectively, from common inflammation.

On better grounds than pathological research, his own clinical observation, supported by the high authorities of Pinel and Esquirol, our author objects to bloodletting. His favorite remedies, in acute mania, are tartar emetic in small doses, occasional laxatives, the tepid bath, cold applications and occasional leeches to the head, and low diet. In a more advanced stage, with a pale face and feeble pulse, he has given wine, valerian, and even cinchona with advantage. He has found colchicum useful both in acute and chronic cases. In a section in this commentary on the crimes which are imputed to insanity, Sir Alexander displays a dread that the wholesome principle of moral and legal responsibility for actions should be lost sight of in Dr. Prichard's doctrine of moral insanity. After much attention to Dr. Prichard's views on this subject, and no inconsiderable observation of the disease to which they refer, we have attained the same conclusion as this very able writer, that the moral sentiments are, equally with the intellectual powers, liable to *derangement* from disease; and we cannot concur in any censure of him for having given his sentiments on the subject freely to the public, considering with him, that "the real facts of a difficult question must be known in their true relation, before a solution can be sought with any prospect of success."* We would remark, however, that in a considerable number of the instances of this moral insanity which have fallen under our observation, intellectual disorder has been associated with it; and we think that such disorder is discernible in some of the cases related by Dr. Prichard in the article we have quoted, particularly in that of the lady, designated A. M——. But we would not deny that cases occur in which the derangement is mainly, if not exclusively, in the moral sentiments. If this truth, and we are convinced that it is one, adds to medical perplexities in a case in which they are already sufficiently abundant, we cannot help it. The clue to the solution of the difficulty must be sought in the comparison of what the patient's feelings are, with what they have been. In a case of this kind, we have seen one who had been the fondest of mothers loathe her children, lamenting bitterly, at the same time, that she did so.

*Syphilis.* The volume concludes with extracts, occupying about eighty pages, relative to the non-mercurial treatment of syphilis, as practised by the late Dr. Fricke in the hospital at Hamburg. The plan of treatment may be summed up in a few words: cleanliness, repose, low diet, and laxative medicine. The diet of the patients in the beginning of their cure consists of four ounces of wheaten bread and about a pint of *soupe maigre*, with some flour mixed in it, three times a day, and at dinner six tablespoonfuls of vegetables are given in addition. The patients are not allowed brandy, wine, or even *water*. If thirsty, barley-water is given them. As soon as the peculiar character of the sores or the complaint begins to disappear, the diet is changed according to the strength and constitution of the patients. In the progress of recovery animal food is allowed; but in the case of females, whose treatment seldom requires more than *three or four weeks'* residence in the hospital, and who require less food than men, the prescribed diet has continued till their dismissal.

The medical treatment is simple. Bloodletting is now employed, only where the patient is of full habit or there is much inflammation. In ordinary cases it is deemed sufficient to give the patient, at first thrice a day, afterwards only once, a tablespoonful of a solution of Epsom salts

*Article on Insanity, (Cyclopædia of Practical Medicine, vol. i. p. 19.)

in fennel water, in the proportion of an ounce and a half of the salt in six ounces of the fluid, called the *English mixture.* We shall not go through the detail of the various local applications employed.

Cases are given in abundance, which prove what every well-informed medical man was already aware of, that syphilis in every form is curable without mercury simply by antiphlogistic means. This, then, is no longer the question. The real subject of inquiry is, ought it to be so treated? Exclusive of the evidence from Dr. Fricke, which properly considered leads, we think, to the same conclusion, but to this we shall subsequently advert, we would say that we have ample reasons for deciding that it ought not. We witnessed for three years in a large hospital, containing always a great number of venereal patients, the simply antiphlogistic treatment of the disease, not a particle of mercury being employed—nay, a great proportion of the patients were treated by ourselves; and we closed the experiment, which as to the question of curability was perfectly decisive, with the conviction that we should have done more justice to our patients had we associated with our antiphlogistic treatment a mild and moderate course of mercury, which conviction subsequent experience has tended to confirm. We argue not for the abuse of the mineral; but such a course as we recommend would, we believe, have abridged the period of treatment, rendered the *sequelæ,* such as eruptions and iritis, much less frequent than we found them, and would rather have spared than injured the constitution of the patients. Sir Alexander is convinced that the method of Fricke will not succeed in private practice, because patients will not submit to the restrictions imposed. We would say further, that it ought not to be followed exclusively in hospital practice.

We have adverted to a reason discernible in Fricke's cases for a less exclusive mode of practice. We find it in the prodigious duration of the treatment. We shall take a few cases in the order in which they stand. Johanna H. was cured of chancre and discharge from the vagina in two months and six days. Lisette Julia M. had a chancre and excoriation; dismissed cured at the end of the sixth week. A case with four chancres on the genitals, and the right tonsil slightly ulcerated and of a deep red colour, cured in two months and fifteen days. In the next case, the patient had undergone treatment prior to admission. Some of the symptoms had disappeared; but a single chancre remained on the glans penis, a line and a half in depth, and six lines wide. The sore at the end of the ninth week from his admission was reduced to the size of a pin's head, when the patient, out of temper from delay, (as well he might be,) departed. He returned eight days after, the ulcer having become larger, and at the end of other three weeks he was at length cured.

Sir Alexander Crichton's explanation of Fricke's success is founded on Liebig's doctrine of metamorphosis by the application of an animal poison. He would say that from the low diet and the purgative medicine employed, the parts of the blood are not supplied which are susceptible of the metamorphosis, the same result being produced as is effected in fever by the annihilation of the desire for food and of the process of chymification. We feel no objection to this explanation; but we would remark that one equally good is offered in the inflammatory character of all the products of the syphilitic virus and the antiphlogistic nature of the course employed.

We now take leave of Sir Alexander Crichton, and, we assure him, with the respect we entertained for him on account of the efforts he has made at his period of life to instruct his brethren—or rather, we should say, his children—enhanced by the value of the lessons he has imparted to them. On some points we have differed from him, and have been free in our expression of this difference; but he has shown himself too candid in declaring his sentiments of others to render it possible that he should be displeased with the display of the same quality in us.

---

